# *Indigo naturalis* as a potential drug in the treatment of ulcerative colitis: a comprehensive review of current evidence

**DOI:** 10.1080/13880209.2024.2415652

**Published:** 2024-10-30

**Authors:** Yu Hu, Liu-lin Chen, Zhen Ye, Lin-zhen Li, Huan-zhu Qian, Ming-quan Wu, Juan Wang, Kai-hua Qin, Qiao-bo Ye

**Affiliations:** a School of Basic Medical Sciences, Chengdu University of Traditional Chinese Medicine, Chengdu, China; b Department of Pharmacy, Sichuan Provincial Orthopedic Hospital, Chengdu, China; c School of Public Health, Chengdu University of Traditional Chinese Medicine, Chengdu, China; d Health Preservation and Rehabilitation College, Chengdu University of Traditional Chinese Medicine, Chengdu, China

**Keywords:** Traditional Chinese medicine, IN, clinical evidence, mechanism, AhR, intestinal microbiota

## Abstract

**Context:**

Ulcerative colitis (UC) is an intractable inflammatory bowel disease that threatens the health of patients. The limited availability of therapeutic strategies makes it imperative to explore more efficient and safer drugs. *Indigo naturalis* (IN) is a traditional Chinese medicine that possesses many pharmacological activities, including anti-inflammatory, antioxidant, and immunomodulatory activities. The treatment potential of IN for UC has been proven by numerous preclinical and clinical studies in recent years.

**Objective:**

This article provides a comprehensive review of the utility and potential of IN in the treatment of UC.

**Methods:**

‘Indigo naturalis’ ‘Qing dai’ ‘Qingdai’ ‘Ulcerative colitis’ and ‘UC’ are used as the keywords, and the relevant literature is collected from online databases (Elsevier, PubMed, and Web of Science).

**Results and Conclusion:**

Indirubin, indigo, isatin, tryptanthrin, and β-sitosterol are considered the key components in the treatment of UC with IN. Both preclinical and clinical studies support the efficacy of IN for UC, especially in severe UC or in those who do not respond to or have poor efficacy with existing therapies. The mechanisms of IN for UC are associated with the aryl hydrocarbon receptor pathway activation, immune regulation, oxidative stress inhibition, and intestinal microbial modulation. However, the clinical use of IN has the risks of adverse events such as pulmonary hypertension, which suggests the necessity for its rational application. As a potential therapeutic agent for UC that is currently receiving more attention, the clinical value of IN has been initially demonstrated and warrants further evaluation.

## Introduction

Ulcerative colitis (UC) is a chronic and nonspecific inflammatory disease of the rectum and colon with unknown etiology (Qiao et al. 2021). Patients often suffer from recurrent episodes of bloody stool, abdominal pain, and diarrhea. The inflammation spreads from mild to extensive colon and may lead to intestinal damage, permanent fibrosis, and even disability as the disease progresses (Wetwittayakhlang et al. 2021). Furthermore, chronic inflammation in the intestine increases the risk of cancer. Patients with UC are approximately 2.4-times more likely to develop colorectal cancer than the healthy population (Rivera et al. 2022). As a poor-healing disease, UC has become a huge public health challenge that threatens the lives of patients. However, the current treatment status remains less than promising. 5-ASA, the first-line choice for UC, is not available for all patients and is limited to moderate to severe UC. Patients with moderate to severe UC usually rely on drugs such as corticosteroids and thiopurines, which have significant toxic effects and may even induce serious adverse events (Li et al. 2024). Therefore, it is of great importance to develop highly efficacious and safer therapeutic strategies for UC.

Some emerging therapies, including biological agents, probiotics, fecal microbiota transplantation, and natural products, have especially gained attention in the treatment of UC (Hu et al. 2021; Dai, Fan, and Wang 2022). Herbal medicine processes the advantages of good curative benefits, high accessibility, and low side effects, which have important clinical values in Asia, particularly in China (Gupta et al. 2022). Indigo naturalis (IN), a blue powdered traditional Chinese medicine (TCM), is made through a complex process from the leaf or stem of *Strobilanthes cusia* (Nees) Kuntze (Acanthaceae), *Persicaria tinctoria* (Aiton) Spach (Polygonaceae), or *Isatis tinctoria* L. (Brassicaceae) (Sun et al. 2021). IN is first applied as a natural pigment. Beginning in the Tang Dynasty, its clinical value has been reported in Chinese medical writings such as *Theory of Medicinal Properties* (Tang Dynasty, 627 A.D.), *Kaibao Materia Medica* (Song Dynasty, 973 A.D.), and *Materia Medica Yanyi* (Song Dynasty, 1116 A.D.) with documented applications in gastrointestinal diseases such as *Classified Materia Medica from Historical Classics for Emergency* (Song Dynasty, 1082 A.D.) and *Compendium of Materia Medica* (Ming Dynasty, 1578 A.D.).

In TCM theory, IN is salty and cold in nature, with the effect of clearing heat and detoxification, expelling fire, and cooling blood. Consequently, IN can be used to treat various diseases caused by heat and toxin, under the guidance of the principle of syndrome differentiation and treatment (Qi-Yue et al. 2020), such as hemorrhage, swelling of the throat, ulcers, pediatric convulsions, and epilepsy (Zhang et al. 2022). The accumulation of dampness, heat, and toxin in the intestine is considered the critical pathological factor causing UC, and the large intestinal damp-heat syndrome and heat-toxin syndrome are two highly prevalent subtypes of UC (Chen, Shen, and Jiang 2022). As a result, IN is frequently used to treat UC based on the TCM theory.

The therapeutic promise of IN for UC has been proven by numerous preclinical and clinical studies. Many studies have also demonstrated numerous activities of IN, such as anti-inflammatory, antibacterial, antiviral, and immunomodulatory roles, suggesting the diversity of its pharmacological mechanisms for UC (Qi-Yue et al. 2020). However, there is a paucity of comprehensive summaries of the efficacy and safety, as well as the precise mechanisms, for the treatment of UC with IN. In this study, ‘Indigo naturalis’, ‘Qing dai’, ‘Qingdai’, ‘Ulcerative colitis’, and ‘UC’ are used as the keywords to search the relevant literature from Elsevier, PubMed, and Web of Science databases without any limitation. After the review of literature, we introduce the effective compounds and their pharmacological properties in IN. Then, the preclinical and clinical evidence is reviewed systematically. Finally, the possible mechanisms of IN in the treatment of UC are summarized based on the available evidence, which aims to confirm its utility and potential in the treatment of UC.

## The main compounds and their pharmacological activities for UC in IN

**Figure 1.**
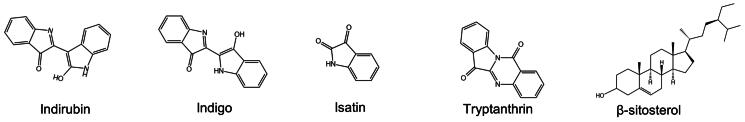
The chemical structures of five components in in.

More than 60 compounds have been isolated from IN to date, including but not limited to indole alkaloids, terpenoids, organic acids, steroids, and nucleosides. IN has been reported to possess biological activities such as anti-inflammatory, antioxidant, antimicrobial, antiviral, antitumor, and immunomodulatory, suggesting its potential in the treatment of various diseases. A detailed review of the chemical compositions and pharmacological effects of IN has been published (Qi-Yue et al. 2020; Sun et al. 2021). Therefore, the ingredients that have therapeutic potential for UC *in vivo* or *in vitro* studies, instead of a holistic review of all components contained in IN, are focused on in this study. Specifically, indirubin, indigo, isatin, tryptanthrin, and β-sitosterol are emphatically introduced (Figure 1).

### Indigo and indirubin

Indigo and indirubin were first isolated from IN in 1978. According to the *Pharmacopeia of the People’s Republic of China* (2020 Edition), at least 2% indigo and 0.13% indirubin have become the minimum criteria for quality control of IN. Therefore, indigo and indirubin have also been considered the main bioactive ingredients of IN. Indirubin is the 3, 2′-bis-indole isomer of indigo, both of which are not contained in the original plants but are produced during the process. It has been reported that the glycosidic bond of indoxyl-3-O-β-d-glucopyranoside is broken in immersion and hydrolyzed to dissociative indoxyl catalyzed by kinases in plant cells and microorganisms. Then, the indoxyl is converted to indoline-3-one in an alkaline environment, to indigo after being oxidized, or to isatin for further synthesis into indirubin (Yu et al. 2021).

The anti-UC activity of indigo and indirubin has been proven by dextran sulfate sodium (DSS) or trinitrobenzene sulfonic acid (TNBS)-induced colitis models in preclinical studies (Yang et al. 2021). Indirubin is the critical anti-colitis substance in IN, which significantly inhibits tumor necrosis factor-alpha (TNF-α), interferon-gamma (IFN-γ), interleukin (IL)-2, and myeloperoxidase (MPO) levels and increases IL-10 levels in the colon (Gao et al. 2016; Tokuyasu et al. 2018). Its anti-inflammatory effect may lie in the inhibition of inflammatory signaling pathways such as mitogen-activated protein kinase (MAPK) and nuclear factor kappa b (NF-κB) (Gao et al. 2018). In addition, indirubin can process immunomodulatory activity by suppressing the infiltration of CD4^+^ T cells and increasing the formation of regulatory T (Treg) cells in the colon (Gao et al. 2016). However, the low water solubility and poor pharmacokinetic properties of indirubin are the main obstacles limiting its clinical application. Notably, research on chemically modified derivatives of indirubin is becoming a new trend, which can be conducive to its potential for treating diseases (Wang et al. 2021).

As an isomer of indirubin, the anti-inflammatory properties of indigo is similar to those of indirubin, including inhibiting the NF-κB pathway and activating the intestinal aryl hydrocarbon receptor (AhR) signaling pathway to produce IL-10 and IL-22, which are the important anti-inflammatory cytokines in UC (Kawai et al. 2017; Yang et al. 2021). Moreover, indigo can accelerate cell scratch closure in the *in vitro* caco-2 cell model, indicating its healing effect on intestinal epithelial injury (Shimizu et al. 2021). Furthermore, indigo has strong antioxidant activity by scavenging superoxide anion in a concentration-dependent manner (Zhao et al. 2017). In addition, indigo can exhibit antioxidant effect through non-enzymatic antioxidant and inhibitory pathways of polymorphonuclear neutrophil infiltration in the ethanol-induced oxidative damage model (Farias-Silva et al. 2007), suggesting its potential in the prevention of excessive oxidative damage in UC.

### Isatin

A portion of isatin that is not converted to indirubin during the process of IN has also pharmacological activities. Similarly, isatin can ameliorate experimental colitis by inhibiting MAPK and NF-κB signaling pathways and alleviating the oxidative damage in colon (Gao et al. 2018). Additionally, the antibacterial activity of isatin is demonstrated by *in vitro* studies. *Staphylococcus aureus*, *Streptococcus pneumoniae*, *Escherichia coli*, and *Klebsiella pneumoniae* are susceptible to isatin, revealing its antibacterial property against harmful microorganisms in the intestine (Chiang et al. 2013).

### Tryptanthrin

Tryptanthrin, also categorized as an indole alkaloid, was first found from IN in 1983. Tryptanthrin can improve DSS-induced colitis by interfering with both the NF-κB and signal transducer and activator of transcription 3 (STAT3) pathways (Wang et al. 2018a). *In vitro*, tryptanthrin can exert anti-inflammatory activity by inhibiting nitric oxide (NO) production by IL-1β (Ozawa et al. 2020).

### β-Sitosterol

β-Sitosterol is a sterol-like compound that was first discovered in 1984 from IN (Sun et al. 2021). In both DSS and TNBS-induced colitis in mice, the expression of TNF-α, IL-1β, and IL-6 is significantly decreased after β-sitosterol administration (Lee, Kim, and Kim 2012; Ding et al. 2019). More importantly, it is confirmed *in vitro* that β-sitosterol can increase the expression of antimicrobial peptides in the intestine, alleviate the colonization of pathogenic microorganisms, and restore the intestinal microbial homeostasis, indicating that β-sitosterol may contribute to the regulation of intestinal microbiota by IN in UC (Ding et al. 2019).

The anti-UC activities, mainly including anti-inflammatory and antioxidant pharmacological functions, of all the above five compounds have been proven, suggesting the potential of IN for UC. Remarkably, some new compounds that may have anti-UC potential are being extracted from IN. The differentiation of Th17 cells and the production of IL-17 play important roles in the development of UC. Lee et al. (2019) have found that indigodole A and indigodole D isolated from IN can inhibit the production of IL-17 in Th17 cells, and indigodole C and cephalandole B can reduce the expression of the *IL17* gene (Lee et al. 2020). In conclusion, the exploration of bioactive ingredients in IN may help to discover more efficient therapeutic agents, which can provide stronger evidence of IN in the treatment of UC.

## The therapuetic potential and possible mechanisms of IN for UC

### The preclinical evidence of in for UC

Numerous preclinical studies supporting the promising role of IN in the treatment of UC have been conducted (Table 1). The intervention of IN and its bioactive compounds (indigo, indirubin, tryptanthrin, isatin, or β-sitosterol) can improve typical symptoms, comprising bloody stool, weight loss, and diarrhea, and decrease the disease activity index (DAI) of DSS-induced colitis, which is the most commonly used UC model (Gao et al. 2018; Ozawa et al. 2020; Sun et al. 2020; Yang et al. 2021). Importantly, IN exerts better efficacy for experimental colitis when comparing 5-ASA as positive controls (Ozawa et al. 2020). In the TNBS-induced colitis model, IN and indigo can also improve the symptoms, DAI score, and pathological lesions of animals (Kawai et al. 2017).

**Table 1.** Preclinical evidence for the treatment of UC with IN and its ingredients.

Study	Animal models	Drugs	Main outcomes	Mechanisms	Conclusions
Yang et al. (2021)	BALB/C mice with DSS	IN Indigo Indirubin	IN, indigo, and indirubin reduced DAI scores, inhibited colonic shortening, improved pathological changes, and restored intestinal mucin expression. IL-1β, IL-6, and TNF-α levels were decreased by IN, indigo, and indirubin. IN could reduce IgG level both in serum and tissue and decrease MPO level in serum. The intestinal microbiota was regulated by IN, indigo, and indirubin.	IN regulated the TLR/MyD88/NF-κB pathway and restored the intestinal microbiota to treat experimental colitis.	IN treated UC by suppressing inflammation and modulating the gut microbiota.
Sun et al. (2020)	SD rats with DSS	IN	IN alleviated rectal bleeding, stool consistency, weight decrease, and reduced the DAI score of rats. The histological score was decreased after IN treatment. The MPO and TGF-β levels were relieved by IN. The dysbiosis of intestinal microbiota was alleviated by IN. IN increased the butyrate acid production and its sensor GPR41/43 level.	IN promoted butyrate production and GPRs expression, leading to an activation of anti-inflammatory signaling cascades in the intestinal mucosa by regulating intestinal microbiota.	IN treated UC by suppressing inflammation and modulating the gut microbiota.
Ozawa et al. (2020)	C57BL/6JJmsSlc mice with DSS or TNBS	IN Tryptanthrin Indigo	IN was effective in improving symptoms (diarrhea and bleeding) and restoring colon histology, and the inflammatory genes (*MMP3*, *PTGS2/* *COX2*, *IL1β*, *IL1R1*) were suppressed. Tryptanthrin maintained the weight of mice but is insufficient in suppressing diarrhea or bleeding. Indigo alleviated bleeding but did not strongly suppress body weight loss or diarrhea.	IN, including its ingredients tryptanthrin and indigo, could exert anti-inflammatory activity via inhibiting NO production induced by IL-1β.	IN treated UC by reducing oxidative stress and inhibiting inflammation.
Liang et al. (2019)	Kunming mice with DSS	IN	The clinical activity score and histopathologic score were decreased after IN administration. IL-6, IL-8, and TNF-α levels were decreased, IL-10 levels was increased by IN. IN restored the dysbiosis of intestinal microbiota.	The therapeutic effect of IN may be linked to the restoration of microbiota immunity, especially the *Turicibacter* and *Peptococcus*.	IN treated UC by restoring gut microbial homeostasis.
Gao et al. (2018)	BALB/c mice with DSS	Indirubin Isatin	Indirubin and isatin could decrease the DAI score and improve the histological damage of mice. Indirubin combined with isatin reduced the TNF-α, IFN-γ, IL-6, iNOS, MPO, and MDA levels and increased the IL-10, SOD, and GSH levels. Indirubin combined with isatin inhibited CD4^+^ T cell infiltration and elevated the Foxp3 expression. The cell apoptosis was suppressed by indirubin and isatin.	Indirubin and isatin could inhibit the NF-κB and MAPK signaling pathway.	IN (indirubin and isatin) treated UC by inhibiting inflammation.
Wang et al. (2017)	SD rats with DSS	IN	IN significantly decreased the DAI score and colonic histopathologic score of rats, the MPO, IL-1α, IL-1β, and IL‑18 levels were reduced by IN. EGF, VEGF, and occludin contents were restored. ALT and AST did not increase after IN treatment.	IN improved the inflammatory response by modulating pro-inflammatory cytokines and improved colonic mucosal damage by repairing tight junction proteins.	IN treated UC by suppressing inflammation.
Kawai et al. (2017)	C57BL/6J mice with DSS or TNBS	IN Indigo	IN and indigo could ameliorate the inflammation and colonic injury (the DAI score, histopathologic score, and colon shorten were decreased) in DSS or TNBS-induced colitis *IL10, IL22* and *Cyp1a1* mRNA expression in mononuclear cells in lamina propria were increased by IN. CD4^+^IL-10^+^ cells and CD32^-^RORγt^+^IL-22^+^ cells were increased by IN or indigo.	IN and indigo ameliorated experimental colitis by activating the AhR signaling.	IN treated UC by modulating the AhR signaling.
Gao et al. (2016)	BALB/c mice with DSS	Indirubin	Indirubin could significantly decrease DAI score and histological score of colitis mice. TNF-α, IFN-γ, IL-2, and MPO levels were decreased, and IL-10 level were increased by indirubin. The infiltration of CD4^+^ T cells were suppressed, and the formation of Foxp3^+^ expressing T cells were promoted by indirubin.	Indirubin could regulate colon inflammation by inhibiting the NF-κB signaling pathway, reducing CD4^+^ T cell infiltration and promoting the generation of Foxp3 expressing Treg cells.	IN (indirubin) treated UC by suppressing inflammation based on the modulation of immune cells and inhibition of inflammatory pathways.

Abnormal immune responses, excessive oxidative damage, intestinal barrier disruption, and microbial disorders play critical roles in the development of UC. IN can intervene in multiple etiologies of UC. Firstly, both the whole IN and extracted compounds are effective in inhibiting the release of pro-inflammatory cytokines and increasing anti-inflammatory cytokines such as IL-10 and IL-22, to balance abnormal inflammation in the colon (Kawai et al. 2017). Secondly, indirubin and isatin have been demonstrated to reduce the levels of inducible nitric oxide synthase (iNOS) and malondialdehyde (MDA) and restore superoxide dismutase (SOD) and glutathione (GSH) levels to relieve oxidative stress (Ozawa et al. 2020). Furthermore, IN can restore the levels of epidermal growth factor (EGF), vascular endothelial growth factor (VEGF), and occludin proteins, thus maintaining the intestinal epithelial barrier (Wang et al. 2017). Finally, IN can ameliorate the dysbiosis of the intestinal microbiota in UC models, which is beneficial to restoring microbial homeostasis (Sun et al. 2020). As a result, IN modulates several key pathological aspects through multi-component, multi-pathway mechanisms to improve UC

Notably, Kei Ozawa et al. (2020) obtain several intriguing results for the therapeutic effects of bioactive compounds in IN. It has been showed that tryptanthrin can maintain body weight but is not significant on hemorrhage, while indigo can improve hemorrhage but is limited to maintaining the body weight of animals. Similarly, the methanol-soluble components of IN only improve body weight but not hemorrhage, while the methanol-insoluble fractions can improve symptoms of diarrhea and hemorrhage in colitis animals. Critically, the combination of two fractions brings out the full anti-UC activity of IN. In addition, the combined administration of indirubin and isatin provides better protection against UC than treatment alone (Gao et al. 2018). These important findings indicate that different components in IN may assume various anti-UC activities and mechanisms. Therefore, the holistic application of IN, or a combination of multiple compounds rather than a single compound, maybe a key means to improve the clinical efficacy of IN.

### The clinical evidence of IN for UC

Extensive of clinical studies support the efficacy of IN for UC (Table 2). Two randomized controlled trials (RCTs) have been conducted in Japan with positive results. Uchiyama et al. (2020) have reported that 23 UC patients have higher clinical response rates, improved albumin levels, and increased Lichtiger indexes compared to those in the placebo group when treated with IN at a dose of 500 mg for a 2-week course. Makoto Naganuma et al. (2018) establish different doses of IN, 500, 1000, and 2000 mg, respectively, and administer a course of treatment twice a day for 8 weeks to UC. The results suggest that IN exhibits an ameliorating effect on UC in a dose-dependent manner. Moreover, the authors also report that the intestinal microbiota structures of patients have approximated those of healthy volunteers after IN treatment, indicating the role of IN in restoring microbial homeostasis. Both RCTs follow strict randomized, double-blinded, and placebo-controlled principles, which enhance the quality of clinical evidence. In addition, in a *post hoc* analysis of their research, Naganuma et al. (2020) find the clinical efficacy of IN in steroid-dependent UC patients or those who have been treated with anti-TNF-α agents, suggesting the clinical value of IN in refractory UC.

**Table 2.** Clinical evidence for the treatment of UC with IN.

Study	Study types	Numbers of patients	Treatments	Outcomes	Safety	Conclusions
Ben-Horin et al. (2024)	RCT	Treatment group: 28, Placebo group: 13	1.5 g IN combined with 1.5 g curcumin one day, for 8 weeks	The clinical remission rate and the clinical response rate were 50% and 8%, 85.7% and 30.7% of treatment group and placebo groups, respectively. The proportions of mayo score reductions of 1 point or more were 75% and 20% in the treatment and placebo groups, respectively. The proportion of treatment and placebo groups with calprotectin reductions of 50% and greater was 46.4% and 15.4%, respectively.	1 patient was reported to have elevated liver transaminases in the treatment group.	IN combined with curcumin could exert better efficacy than placebo for UC.
Yanai et al. (2023)	Prospective study	88 UC patients	2 g IN combined with 2 g curcumin for 60 patients, 2 g IN combined with 3 g curcumin for 15 patients, 0.5 g IN combined with 2 g curcumin for 13 patients	46.5% patients achieved clinical remission, and 60.2% patients achieved clinical response. 7 patients achieved corticosteroid-free remission of which on corticosteroids treatments at baseline. Among 43 biologics/small molecules experienced patients, the clinical remission was achieved in 39.5% and clinical response in 58.1%.	4 patients had mildly elevated transaminases, all of which recovered subsequently. 2 patients experienced self-limiting headaches.	Combination of IN and curcumin improved efficacy and safety for UC.
Ben-Horin et al. (2022)	Case reports	2 UC patients	2.5 g curcumin combined with IN one day for a severe 24-year-old male UC patients refractory to cyclosporine and corticosteroids. 2 g curcumin combined with IN one day for a 59-year-old female with extensive UC not responding to maximal oral + topical 5-ASA and corticosteroids.	The rectal bleeding was rapidly relieved and achieved clinical remission in few weeks for the male patients. Only minimal residual inflammation was observed by endoscopies for 12 weeks. The clinical recession was induced rapidly for the female patients, and the mucosal healing was performed by endoscopies performed after 2 and 5 months. The patient maintained clinical remission for 49 months.	No adverse events were reported.	Combination of IN and curcumin improved efficacy and safety for UC.
Saiki et al. (2021)	Open-label, dose-escalation study	11 UC patients	250 mg or 750 mg oral IN twice a day	91% patients achieved clinical response. The Mayo endoscopic subscore of patients deceased from 2.3 to 1.3 and mean Ulcerative Colitis Endoscopic Index of Severity scores deceased from 8.8 to 5.5	9 patients reported adverse events. The high frequency of adverse events was mild and transient abdominal pain, headache, or flatulence. 1 patient had two serious adverse events, orthostatic hypotension and sepsis, were unrelated to IN in the judgement of the investigator.	IN was effective for UC with no significant adverse effects.
Uchiyama et al. (2020)	RCT	Treatment group: 23, Placebo group: 19	IN powder, 500 mg once a day, for 2 weeks	The albumin and Lichtiger index of treatment group were significantly improved after treatment. Compared with placebo group, patients in treatment group had a high Lichtiger index and response rates.	Mild headaches were observed in 5 patients. No serious adverse events were reported.	IN could exert better efficacy than placebo for UC.
Yoshimatsu et al. (2020)	Prospective study	10 UC patients	1 IN capsule of IN per day for 4 weeks through the rectum.	The clinical remission and mucosal healing rates were 30% and 40% after treatment. The Mayo score was significantly improved from 7.7 to 4.9 and the partial Mayo score significantly improved from a mean of 5.2 to a mean of 2.9.	1 patient developed mild anal pain as a therapy-related adverse event.	4 weeks of IN suppository could be tolerated by UC patients.
Urushikubo et al. (2019)	Case reports	14 UC patients	With 0.5 g or 1.0 g or 2.0 g IN for 2, 5, 7 patients, respectively.	5 patients achieved clinical response and 4 patients achieved clinical remission. Mayo endoscopic subscores decreased from 2 (2–3) to 1 (1–2); rachmilewitz endoscopic index decreased from 7 (5.5–11) to 3 (1–7); UC endoscopy index of severity decreased from 3 (3–4.5) to 1 (0.5–3.5).	1 patient developed acute right-sided colitis with wall-thickening and edematous change after she increased IN dose.	Short-term application of IN was effective for UC without serious adverse events.
Suzuki et al. (2018)	Case reports	20 UC patients	Patients purchased IN powder for their own use and took 1 g oral dose, twice a day.	The response rate was 90% after IN treatment.	5 patients had mild headache after several months of IN administration with disappeared after a half-dose reduction. 2 patients showed a mild and reversible elevation of transaminase level.	Long-term use of IN maintained efficacy against UC.
Naganuma et al. (2018)	RCT	Treatment group: 64, Placebo group: 22	23, 20, and 21 patients received daily doses of 0.5, 1.0, and 2.0 g of IN, twice a day for 8 weeks, respectively.	The response rates had s significant dose-dependent linear trend for treatment group, 1.0 g or 2.0 g IN groups had a significantly better clinical remission compared with placebo group. Mayo score and serum albumin levels of all treatment groups were improved compared with placebo group. The microbiota composition of treatment group patients was similar to that of healthy volunteers.	The incidence of therapy-related adverse events of placebo group, 0.5 g IN group, 1.0 g IN group and 2.0 g IN group were 14%, 25%, 36%, 29%, respectively.	IN could exert better efficacy than placebo for UC.
Sugimoto et al. (2016)	Prospective Study	20 UC patients	Four 250-mg IN capsules, twice a day for 8 weeks.	The overall clinical response rate was 72%. The overall clinical remission rate was 33% after treatment. The average Mayo scores were improved from 7.9 to 2.7. The serum CRP and albumin levels of patients were significantly improved with fecal immunological quantitative test.	No patient had a severe exacerbation requiring hospitalization over the 8-week period nor did any patient start biologics. 2 patients developed reversible liver dysfunction.	Oral IN was effective for inducing remission in moderate UC patients and could be tolerated.
Suzuki et al. (2013)	Retrospective observational study	9 UC patients	Patients took 1 g of IN orally twice a day.	The average clinical activity index scores decreased from 8.3 ± 2.4 to 2.4 ± 3.4. The endoscopic Matts grade decreased from 3.4 ± 0.5 to 2.2 ± 0.8 in 5 patients.	2 patients developed mild headaches which could be disappeared after reduction IN dose.	IN exerted clinical and endoscopic efficacy in UC patients who failed to respond to conventional medications.

The clinical efficacy of the combination medications based on IN for UC has also been approved by some emerging trials. A RCT conducted by Ben-Horin et al. (2024) suggests the curcumin, an TCM compound with broad pharmacological activities, can significantly improve the clinical response rates and clinical remission rates in patients who have failed or are intolerant to at least the current 1 UC treatment when used in combination with IN for 8 weeks course. Moreover, 15 patients who respond to this therapy continue to receive more 8 weeks of treatment, and the result suggests that 14 patients maintain the clinical response, indicating the efficacy of the therapy for long-term use. Similarly, a retrospective study contained 88 patients which have no or partial response to biologics/macromolecules conducted by Yanai et al. (2023) suggests that 46.5% of patients achieve clinical remission and 60.2% of patients achieve clinical response. Of the 9 patients who underwent endoscopy, 8 achieve endoscopic improvement and 4 achieve endoscopic remission. Moreover, Ben-Horin et al. (2024) report that two severe UC patients who do not respond to corticosteroids achieve rapid clinical remission after the treatment of curcumin combined with IN, and the endoscopy also indicates its efficacy. Taken together, the IN-based medication may be an effective treatment option for severe UC, especially in patients who do not respond to corticosteroids or biologics.

### The possible mechanisms of IN in the treatment of UC

#### Intervention of immune cells and inflammatory signaling pathways

Immune cells, inflammasomes, and inflammatory signaling pathways are contributors to intestinal inflammation (Ramos and Papadakis 2019). CD4^+^ T cells can differentiate into various T cells mediated by cytokines, including Th1 cells induced by IFN-γ and IL-2, and Th2 cells induced by IL-4 (Bing et al. 2017). TNF-α and IFN-γ act as effectors of Th1 cells and play pro-inflammatory roles in the pathogenesis of UC (Gao et al. 2016). In contrast, IL-10 is an important anti-inflammatory cytokine in intestinal inflammation. Therefore, the Th1/Th2 imbalance is involved in the development of UC. Indirubin can inhibit CD4^+^ T cell infiltrations in the colonic mucosa, further downregulate Th1-type cytokine levels, and upregulate Th2 cytokine levels to regulate the Th1/Th2 imbalance. Another IL-10-secreting T cell, Fxop3^+^ Treg cells, are also induced to differentiate after indirubin intervention, thus exerting anti-inflammatory effects (Gao et al. 2016).

In addition to the involvement of immune cells, the aberrant activation of inflammatory signaling pathways is conductive to chronic intestinal inflammation. Among them, the NF-κB p65 protein is highly expressed in intestinal mucosal epithelial cells, crypt epithelial cells, and lamina propria monocytes in UC patients (Lu and Zhao 2020). Notably, both the application of IN and indigo or indirubin can inhibit colonic NF-κB signaling pathway activation *in vivo* by affecting p65 protein and the inhibitor of κB-α(IκBα) activities, which can reduce the secretion of its effector cytokines, TNF-α, IL-1β, and IL-6 (Gao et al. 2016; Yang et al. 2021). Moreover, the activation of the MAPK pathway is associated with chronic inflammation in UC. p38 MAPK, an isoform of MAPK, its phosphorylation has been found to increase significantly in the nucleus of immune effector cells in the mucosal crypt of UC patients (Dahan et al. 2008). The MAPK signaling pathway promotes the M1 macrophage activation, which releases pro-inflammatory cytokines and reactive oxygen species (ROS) to exacerbate inflammatory damage during UC development (Jing, Wang, and Xu 2019). The combination of indirubin and isatin can significantly decrease the phosphorylation of p38 MAPK, c-Jun N-terminal kinase, and extracellular regulated protein kinases, suggesting its extensive inhibitory activity for the MAPK pathway (Gao et al. 2018). Thus, the regulating effects on inflammatory mediators are also a critical mechanism of IN for treating UC.

#### Inhibition of oxidative stress

Oxidative stress is another necessary risk factor in the pathogenesis of UC (Ma et al. 2023). The production of excess ROS and reactive nitrogen species (RNS) in the intestine, which has exceeded the buffering capacity of the antioxidant defense in the body, leads to lipid peroxidation, intestinal mucosal barrier damage, and bacterial translocation (Wang et al. 2016). Additionally, ROS can act as a second messenger to activate intracellular inflammatory signaling pathways, which participate in chronic intestinal inflammation (Wang, Grivennikov, and Karin 2013)

MPO, nitric oxide synthase (NOS), and cyclooxygenase (COX) can promote endogenous ROS and RNS production by catalyzing chemical reactions (Tian, Wang, and Zhang 2017). MPO is significantly expressed in the inflamed mucosa of UC patients, and COX-2 is also closely associated with colitis (Konturek et al. 2005; Ullman and Itzkowitz 2011). Similarly, inducible nitric oxide synthase (iNOS), an inducible type of NO production, is also widely expressed in mucosal inflammation (Cross and Wilson 2003). After the administration of IN or extracted indigo, indirubin, tryptanthrin, and isatin, the gene expression of *COX2* is inhibited, MPO and iNOS levels are reduced, and NO content is decreased, suggesting that IN and its active compounds can reduce oxidative stress by decreasing ROS and RNS production (Gao et al. 2018; Ozawa et al. 2020; Sun et al. 2020; Yang et al. 2021).

Enhancing the body’s antioxidant defense is another crucial strategy for combating oxidative stress. SOD is an intracellular enzymatic antioxidant that can reduce ROS levels by catalyzing the reduction of superoxide to hydrogen peroxide (Kaur and Benov 2020). GSH, on the other hand, is an intracellular, non-enzymatic antioxidant that can form an antioxidant barrier in the intestinal mucosa (Tian, Wang, and Zhang 2017). The application of indirubin and isatin can significantly restore SOD and GSH levels in the DSS-induced UC model (Gao et al. 2018). Further *in vitro* research reveals that both indigo and indirubin can modulate the nuclear factor erythroid 2-related factor 2-related antioxidant processes to inhibit excess ferroptosis (Yokote et al. 2023). Therefore, the relief of oxidative stress damage by regulating the oxidative/antioxidant balance in the intestine is also one of the important mechanisms of IN.

#### Regulation of the intestinal microbiota

Differences in intestinal microbiota between UC patients and the healthy population are significant, as manifested by decreased microbial diversity, reduced abundance, and changed abundance of specific groups of organisms. IN can help to restore intestinal microbial homeostasis in experimental colitis (Table 3). The imbalance of the Firmicutes/Bacteroidetes ratio due to decreased *Firmicutes* abundance and increased *Bacteroidetes* abundance at the phylum level is a characteristic feature of microbial dysbiosis in IBD patients (Walker et al. 2011). After the intervention of IN, the abundance of *Firmicutes* is restored, and the abundance of *Bacteroidetes* is decreased (Adachi et al. 2017; Liang et al. 2019; Sun et al. 2020). At the genus level, some probiotic bacteria, such as *Lactobacillus*, *ruminococcus_1*, *ruminococcaceae_UCG-005*, *norank_f_Erysipelotrichaceae*, *Butyricicoccus*, *Bifidobacterium,* and *Turicibacter*, are recovered in abundance after IN treatment. On the contrary, the harmful bacteria such as *Escherichia-shigella*, decreases in abundance after IN application. In conclusion, these research projects demonstrate the potential of IN to regulate host intestinal microbial homeostasis in UC.

**Table 3.** Intestinal microbial alterations mediated by IN.

Study	Diversity	Phylum level	Family level	Genus level
Yang et al. (2021)	NR	*Proteobacteria* (−)	NR	*Lactobacillus* (+) *Streptococcus* (−) *Desulfovibrio* (−)
Sun et al. (2020)	Alpha diversity (+)	*Bacteroidetes* (−) *Actinobacteria* (+) *Firmicutes* (+)	*Bifidobacteriaceae* (+) *Ruminococcaceae* (+)	*Ruminococcus_1* (+) *Ruminococcaceae_UCG-005* (+) *norank_f_Erysipelotrichaceae* (+) *Butyricicoccus* (+) *Bifidobacterium* (+)
Liang et al. (2019)	Alpha diversity (+)	*Firmicutes* (+) *Bacteroidetes* (−) *Proteobacteria* (−)	NR	*Turicibacter* (+) *Eubacterium_nodatum_group* (+) *Peptococcus* (−) *Gemella* (−) *Family_XIII_UCG_001* (−)
Adachi et al. (2017)	NR	*Firmicutes* (+) *Bacteroidetes* (−)	*Lachnospiraceae* (+) *Ruminococaceae* (+) *Lactobacillaceae* (+) *S24-7* (−)	NR

#### Modulation of AhR pathway

**Figure 2.**
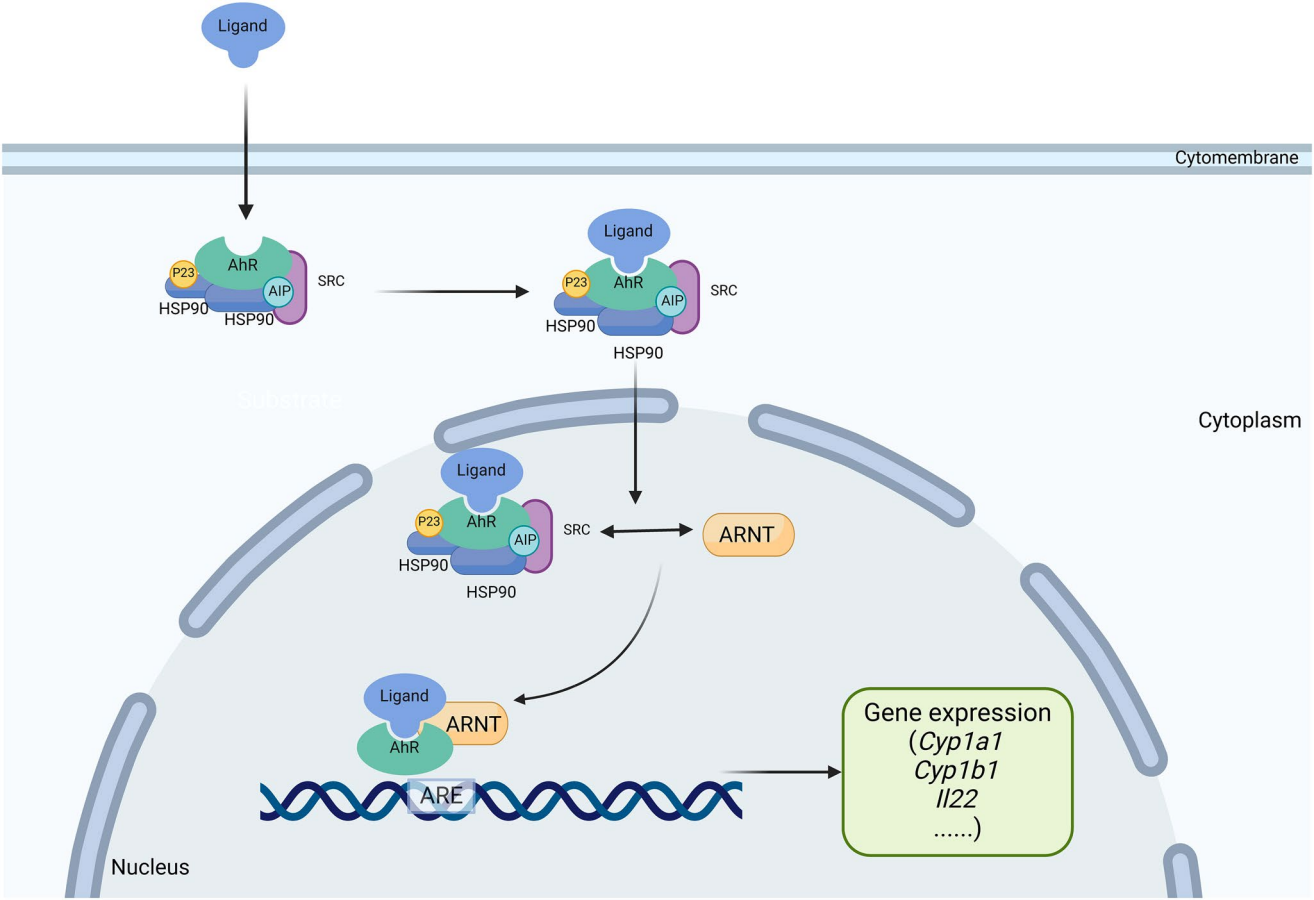
Schematic diagram of the activation process of AhR pathway.

The activation of the AhR pathway by indirubin and indigo as ligands to induce downstream immunomodulatory activity is considered the key mechanism of IN in the treatment of UC (Adachi et al. 2001). AhR is a member of the basic helix-loop-helix/Per-Arnt-Sim family proteins that are widely expressed in vertebrate cells (Kewley, Whitelaw, and Chapman-Smith 2004). AhR is located in the cytoplasm. When it is inactivated, it will form a complex multimer with the 90-kDa heat shock protein (HSP90), the AhR-interacting protein (AIP, also known as XAP2 or Ara9), the cochaperone p23, and the c-SRC protein kinase (Gutiérrez-Vázquez and Quintana 2018). When bound to a ligand, AhR in the cytoplasmic matrix translocates to the nucleus, dimerizes with the AhR nuclear translocator, interacts with the xenobiotic response element (XRE) in the promoter region of the target gene, initiates transcription of target genes such as the xenobiotic metabolic enzyme cytochrome P450 1A1 (*CYP1A1*), and finally induces numerous cascade reactions downstream (Hankinson 1995) (Figure 2).

AhR has been recognized as a key modulator of the host immune and inflammatory systems (Neavin et al. 2018). AhR pathway often plays a protective role in intestinal inflammation (Pernomian et al. 2020). Genome-wide association research has identified *AHR* as a susceptibility locus for IBD (Liu et al. 2015). It is reported that *AHR* expression is significantly lower in the intestinal tissues of IBD patients compared to healthy individuals (Monteleone et al. 2011). Also, mice lacking the *AHR* gene exhibit more severe experimental colitis after DSS induction compared to wild-type mice (Wang et al. 2018b). Moreover, after advanced treatment with the classical AhR ligand, 2,3,7,8-tetrachlorodibenzo-*p*-dioxin, mice suffer mild colonic inflammation even after administration by DSS (Takamura et al. 2010). These findings demonstrate the important regulatory effect of AhR pathway in UC.

Indeed, the activation of the AhR pathway exerts an ameliorative effect on UC through different mechanisms due to its widespread expression in various immune and non-immune cells in the intestine. Disturbances of the immune system and excessive inflammatory responses have irreplaceable roles in the pathogenesis of UC (Ungaro et al. 2017). AhR is widely involved in the intrinsic and adaptive immunity of the intestine by affecting immune cells. Innate lymphoid cells (ILCs), a cell population including NK cells, ILC1, ILC2, ILC3, and regulatory ILCs, are widely present in the mucosal barrier and influence the innate immunity of host (Li et al. 2018b). All subsets of ILCs can be found in the intestine, and ILC2 participates in the expression of IL-5 and IL-13, two pro-inflammatory cytokines (Li et al. 2018b). The mRNA expression of *IL13* shows a significant increase in rectal biopsies from UC patients (Inoue et al. 1999). The activation of the AhR pathway can promote host intestinal immune homeostasis from pathogenic bacteria by suppressing intestinal ILC2 and promoting ILC3 function (Li et al. 2018a). Meanwhile, NKp46 and lymphoid tissue inducer-like subsets are two major IL-22-producing subsets of ILCs. IL-22 is important for suppressing abnormal intestinal immunity by promoting the survival and proliferation of epithelial cells and secreting antimicrobial peptide production (Sonnenberg, Fouser, and Artis 2011). It is shown that the AhR pathway is essential for IL-22 production by ILCs, and its deletion affects the differentiation of IL-22-producing ILC subsets in the mouse intestine (Lee et al. 2011; Zelante et al. 2013). In conclusion, ILCs are possibly central to IBD pathogenesis, and the key regulatory properties of the AhR pathway in immunomodulation are increasing in prominence.

Macrophages and dendritic cells are also important members of intestinal intrinsic immunity. In the development of IBD, microbial exposure stimulated by risk factors, particularly epithelial barrier dysregulation, can stimulate macrophage activation, and the engulfment of microbes by phagocytosis will occur, which further produces higher levels of IL-6, IL-23, IL-12, and TNF-α to promote inflammation (Chang 2020). Dendritic cells, also known as antigen-presenting cells, can provide antigen to primary T cells after antigen capture and then participate in T cell differentiations to control T cell responses and activate adaptive immune pathways (Guermonprez et al. 2002; Chang 2020). The effects of the AhR pathway on macrophages and dendritic cells have been demonstrated, including influence on macrophage polarization and interference with antigen presentation by dendritic cells (Bankoti et al. 2010; Shinde et al. 2018). However, it remains unclear about the specific effects and mechanisms of the AhR pathway on macrophages and dendritic cells in the intestine. Notably, it has shown that mice with *AHR* deletion in the intestinal lamina propria macrophages and dendritic cells are more susceptible to DSS-induced colitis, which confirms the effects of the AhR pathway on the involvement of macrophages and dendritic cells in intestinal lamina propria immunity (Chng et al. 2016).

AhR pathway can also protect the intestinal barrier and maintain intrinsic immunity by affecting non-immune cells. The intestinal barrier, constructed by intestinal epithelial cells (IECs) and the mucus barrier, is the first defense lines against pathogenic microbial invasion. The deficiency of intestinal barrier function is thought to emerge before UC and is strongly implicated in the pathogenesis of UC (Kobayashi et al. 2020). The rapid regeneration of IECs is driven by the proliferation of intestinal stem cells in the crypt (Barker et al. 2007), but mice with *AHR*-specific deficiency in IECs cannot maintain the integrity of the epithelial barrier against bacterial infection due to impaired intestinal stem cells differentiation and proliferation (Metidji et al. 2019). Mucin secreted by the goblet cells is the necessary source of mucus. The deficiency of the *AHR* gene leads to the loss of goblet cells and reduced expression of mucin 2 in mice (Yin et al. 2019). Thus, AhR activity is also essential for the maintenance of the intestinal barrier.

Except for ILCs, T cells, another immune cells are involved in intestinal adaptive immunity, also contribute to the expression of the anti-inflammatory cytokine IL-22 in the intestine (Mizoguchi et al. 2018). Various subtypes of T cells, including CD4^+^ Th17 cells and CD4^+^ Th22 cells, can induce IL-22. In addition, IL-17 is one of the major effector cytokines produced by Th17 cells and can promote inflammation in the intestine (Eyerich, Dimartino, and Cavani 2017). It is reported that Th17 cells are an important source of IL-22 expression in mice. However, only a limited number of Th17 cells can express both IL-22 and IL-17 in humans (Eyerich, Dimartino, and Cavani 2017). In contrast, Th22 cells in humans, which can express IL-22 but not IL-17, play a protective role in intestinal inflammation. The activation of the AhR pathway influences Th17 cells in separate aspects (Qiu et al. 2013). Firstly, the absence of *AHR* limits the production of IL-22 induced by ILCs, which further leads to an increased abundance of commensal segmented filamentous bacteria that stimulate Th17 cell responses, resulting in increased Th17 cell expression and IL-17 secretion, which reflects an indirect effect of the AhR pathway on Th17 cells. Secondly, the AhR pathway can promote the differentiation of Th17 cells into IL-10-producing Treg type 1 cells that no longer express IL-17 but IL-10 during pathogenic microbial infections (Gagliani et al. 2015).

Another T cell subpopulation, Treg cells, also play an important role in colonic inflammation by expressing anti-inflammatory cytokines, such as IL-10 (Coombes et al. 2005). A dysregulation of Th17/Treg balance, with a decrease of Treg cells and an increase of Th17 cells in the colonic mucosa, is a typical manifestation of colonic inflammation in UC patients (Gong et al. 2016). It is proven that AhR expression is significant in intestinal Treg cells in steady state (Ye et al. 2017). However, the specific absence of the *AHR* gene in Treg cells leads to an obvious reduction of Treg cells in the intestine, which is linked to the fact that AhR can promote the intestinal homing of Treg cells (Ye et al. 2017). In summary, the activation of the AhR pathway contributes to suppressing the overactive Th17 response, which is necessary to regulate intestinal inflammation in UC.

**Figure 3.**
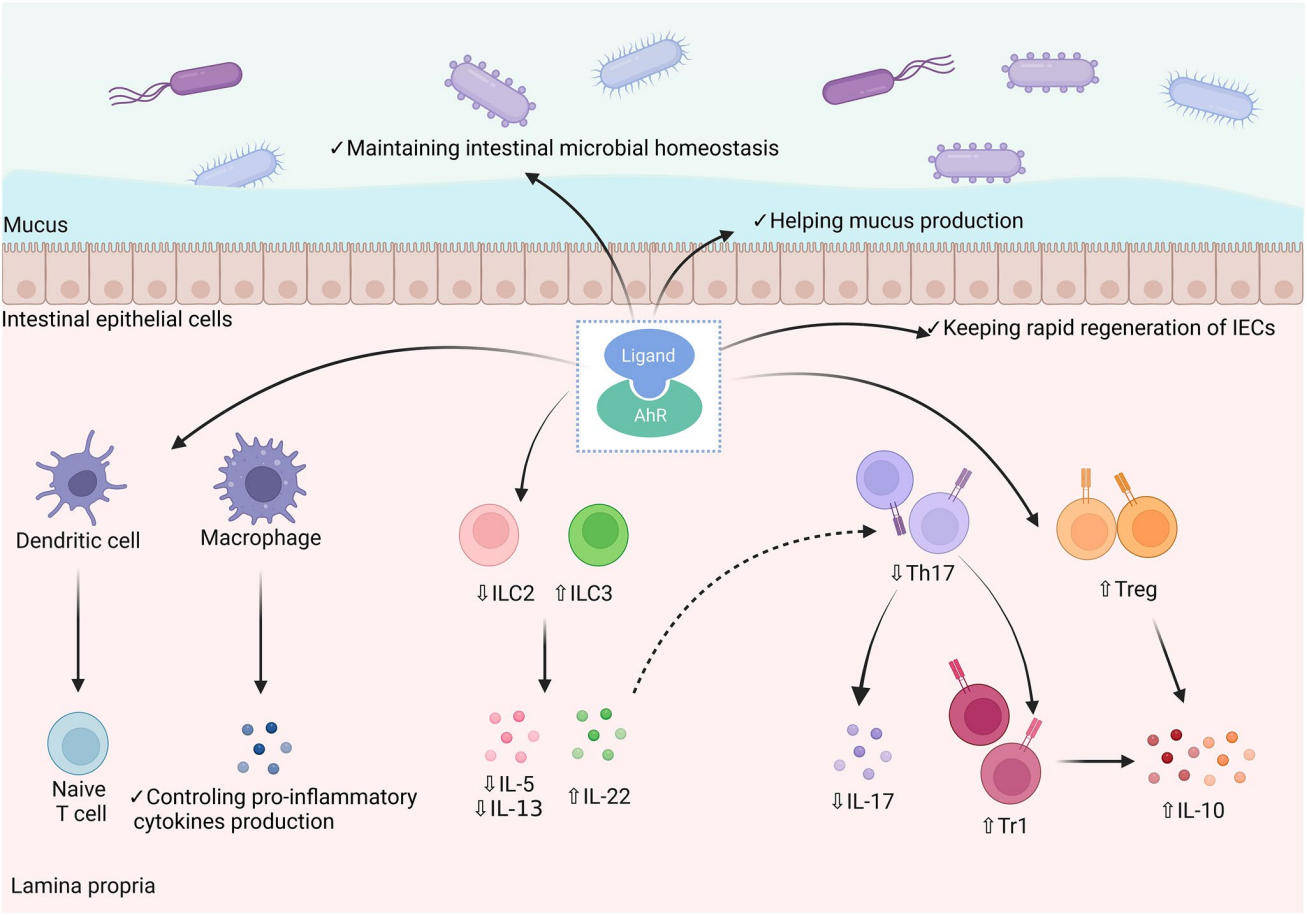
The protective role of AhR pathway activation in intestinal inflammation. AhR is widely expressed in immune and non-immune cells of the intestine. The AhR pathway activation by binding to ligands affects macrophage activation and antigen presentation by dendritic cells to reduce the secretion of inflammatory mediators. In adaptive immunity, AhR pathway activation reduces the release of IL-5 and IL-13 by regulating the ILC2/ILC3 balance and promotes the expression of IL-22. In addition, the AhR pathway can suppress intestinal inflammation by restoring the Th17/Treg balance to reduce IL-17 expression and increase IL-10 expression. Moreover, the AhR pathway can regulate the intestinal barrier and restore microbial homeostasis.

Another non-negligible effect of AhR on colonic inflammation lies in its close association with the intestinal microbiota. The reduction of microbiota diversity and altered composition structure are the main features of microbiota dysbiosis in UC patients (Sokol et al. 2017). It has been demonstrated that the intestinal microbiota can be affected by AhR pathway in preclinical studies. Wild-type mice receiving normal chow has lower proportions of *Firmicutes* and higher proportions of *Bacteroidetes* in the small intestine compared to *AHR*-deficient mice receiving chow without potential AhR ligands (Korecka et al. 2016). Similarly, the *AHR* deficiency significantly affects the microbial composition in the cecum of mice, and these alterations are associated with more severe intestinal inflammation (Murray et al. 2016). Additionally, studies based on AhR signaling activation also support these findings. Exogenous AhR ligand uptake can alleviate colonic inflammation, and improve microbiota dysbiosis, or restore mouse intestinal microbes to normal levels through IL-22-induced pathways (Busbee et al. 2020). Taken together, the AhR pathway in the intestine can improve intestinal inflammation and alleviate UC through multiple effects (Figure 3).

The activation of AhR is identified as the key mechanism of IN in the treatment of UC. Indigo and indirubin exhibit potent AhR ligand activity *in vitro*, even comparable to that of 2,3,7,8-tetrachlorodibenzo-p-dioxin. Likewise, the activation of AhR ligands *in vivo* by indigo and indirubin has been demonstrated (Sugihara et al. 2004). Interestingly, Kawai et al. reveal that indigo and indirubin can ameliorate DSS-induced mouse colitis in an AhR pathway-dependent manner (Kawai et al. 2017). The mononuclear cells isolated from mice show a significant increase in Cyp1A1, a downstream factor of AhR. IL-10 and IL-22, the anti-inflammatory cytokines released by immune cells affected by AhR, can be stimulated by IN and indigo. Furthermore, IN and indigo are only involved in AhR pathway in the intestinal mucosa and do not affect the mesenteric lymph nodes or spleen, suggesting that the activation of AhR by IN seems to present intestinal specificity (Kawai et al. 2017). Yokote et al. (2023) have found that the expression of Cyp1A1 in the rectal mucosa of UC patients receiving IN treatment is significantly increased, with indirubin detected in the rectal mucosa of patients. This finding directly provides clinical evidence for IN and its main active ingredient, indirubin, to treat UC by activating the AhR pathway.

Considering the beneficial effects of the AhR pathway on intestinal microbiota, it is reasonable to assume that IN has an indirect influence on intestinal microbiota through the AhR pathway. Sun et al. (2020) find that IN exerts therapeutic effects on experimental colitis in a microbiota-dependent manner when using an antibiotic-induced germ-free mouse model. They further demonstrate that the specific mechanism may be related to the increase of butyrate, a short-chain fatty acid beneficial to intestinal homeostasis, induced by IN. There is a bidirectional interaction between the AhR pathway and the intestinal microbiota. Tryptophan in humans can be metabolized mainly through the kynurenine or serotonin pathway (Gao et al. 2018). The intermediate metabolites produced *via* the kynurenine pathway, including kynurenic acid, *O*-aminobenzoic acid, cinnamic acid, and flavoracil, have been shown to be endogenous ligands of AhR (Romani et al. 2014). The kynurenine pathway can be induced by the intestinal microbiota. Moreover, approximately 4-6% of tryptophan can be metabolized by the intestinal microbiota into indole, indican, tryptamine, and skatole, as well as indole acid derivatives (Yokoyama and Carlson 1979). Among them, indole and indolic acid derivatives are also ligands that can activate the intestinal AhR pathway (Gao et al. 2018). Zelante et al. (2013) have reported that *lactobacillus* can activate AhR pathway by interfering with tryptophan metabolism and induced IL-22 secretion by ILC3, to maintain intestinal mucosal immunity.

Obviously, there is an intricate interplay between the microbiota, AhR pathway, and host intestinal immune system (Rooks and Garrett 2016). The precise mechanism underlying the therapeutic effects of IN in UC is yet to be determined. It is unclear if the efficacy is primarily due to the direct activation of the AhR pathway or if it is mediated through indirect effects on the intestinal microbiota. In any case, AhR may be the core treatment target of IN on UC based on its wide range of regulating effects.

## Conclusions and prospects

**Figure 4.**
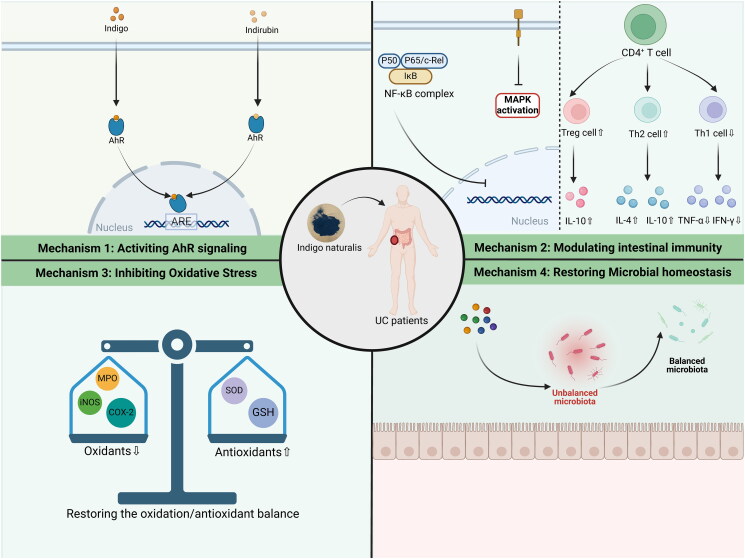
The possible mechanisms of IN in the treatment of UC.

IN has significant clinical value in various diseases under the guidance of TCM theory, which has been used for thousands of years in China. Available evidence suggests that IN may be a promising therapeutic strategy for UC. The multi-component, multi-pathway, and multi-mechanism characteristics of IN determine its ability to intervene in critical pathogeneses, which include inflammatory responses, immune disorders, oxidative stress, intestinal barrier disruption, and intestinal microbial disorders in UC (Figure 4). Moreover, the efficacy of IN for UC, even refractory UC, has been demonstrated by clinical studies, which is certainly exciting.

However, some pressing issues that limit the clinical application of IN in UC need to be addressed. Firstly, the theory of syndrome differentiation and treatment is the basic principle of TCM for application, which means that it is rare to use a single TCM in clinical practice. On the contrary, the synergistic application of various TCMs has the potential to enhance therapeutic efficacy and concurrently attenuate the risks associated with toxic side effects and adverse events. IN has the nature of cold in TCM theory, it may affect the functions of the spleen and stomach in patients with long-term use, causing the occurrence of adverse events such as gastrointestinal symptoms, which have also been reported in clinical trials. The emergence of serious adverse events, including abnormal liver function and pulmonary hypertension, suggests that it is difficult to guarantee the safety of IN alone in the treatment of UC. Therefore, IN should be applied rationally to UC patients under the guidance of medical professionals. UC patients who are undergoing IN therapy should maintain rigorous health monitoring and reduce dosage or withdraw IN when adverse events occur since the majority of adverse events are reversible. Moreover, strict patient management procedures are also necessary for clinicians to minimize the risks associated with IN.

Secondly, the bioactive components and molecular mechanisms of IN for UC have not been fully revealed, which is manifested in two aspects. On the one hand, a few compounds, including indigo, indirubin, isatin, and tryptanthrin, have been focused on in various studies, the characterization of other bioactive constituents with significant pharmacological properties in IN remains an area that requires further investigation. On the other hand, it is still unclear what specific mechanism of UC is regulated by IN. The AhR pathway has multiple regulatory roles in the intestine, so it remains an open question whether the multi-mechanism properties of IN in UC are AhR-dependent. Considering the AhR pathway’s influence on intestinal immune cells and inflammatory signaling pathways, it is still unclear if the modulatory properties of IN on inflammatory mediators are mediated through an AhR-dependent pathway. Further research is required to ascertain whether the activation of the AhR pathway correlates with the influence of IN on the intestinal microbiota, given the established bidirectional relationship between the AhR pathway and the intestinal microbiota.

Lastly, safety is also a necessary indicator of clinical treatment strategies. Noteworthy, Yoshimatsu et al. (2020) have evaluated the therapeutic efficacy of IN in a cohort of 10 UC patients *via* enema administration, resulting in a 40% rate of mucosal healing, as well as significant improvements in both total and partial Mayo scores. Nevertheless, one patient suffers therapy-related mild anal pain during treatment, which led to a question that must be addressed, the safety of IN. The occurrence of adverse events by IN is reported in several clinical trials, although it is effective in reducing symptoms, disease activity scores, and corresponding laboratory indices in UC patients. In the RCT by Naganuma et al. (2018), the use of 500, 1000, and 2000 mg doses of IN results in adverse events in 25%, 36%, and 29% of patients, respectively, with higher rates than that of placebo groups. Urushikubo et al. (2019) report a case of acute inflammation of the right colon in which the patient increases the dose of IN on his own. Naganuma et al. (2019) have investigated 877 Japanese UC patients who have used IN and find that the frequencies of hepatic dysfunction, gastrointestinal symptoms, headache, and pulmonary hypertension are 40,21,13, and 11, respectively. Pulmonary hypertension has become the most concerning adverse event of IN due to its inherent danger. Misumi et al. (2019) report a case of a UC patient who develops pulmonary hypertension after 22 months of IN application and starts to recover after 3 months of IN discontinuation. Kubota K et al. (2023) report that a UC patient with a history of dyspnea begins experiencing exertional dyspnea for 8 months with the diagnosis of pulmonary hypertension after IN treatment, and the symptoms improves after the discontinuation of IN and the application of targeted treatment. Likewise, Inoue et al. (2023) report a case where a UC patient who has been taking IN for 5 years develops drug-induced pulmonary hypertension. The symptoms also improve rapidly after discontinuing IN for 5 days and being discharged from the hospital 10 days later. The above case reports suggest that a history of long-term IN use and underlying respiratory diseases in UC patients may increase the risk of developing pulmonary hypertension. A prospective study by Orihara et al. (2020) has found an association between increased duration of IN and elevated pulmonary artery systolic pressure in patients. All the findings support the strong link between IN and pulmonary hypertension. Therefore, the continued reports of adverse events induced by IN still imply that it is of great importance to take steps, such as monitoring of the health status of patients, optimizing dosage and periodicity of IN administration, and strengthening research on the safety of IN, to minimize possible adverse events associated with IN.

Taken together, as a potential therapeutic agent for UC that is currently receiving more attention, the clinical value of IN has been initially demonstrated and warrants further evaluation.
